# Impact of body mass index in therapeutic response for HER2 positive breast cancer treated with neoadjuvant targeted therapy: a multi-center study and meta-analysis

**DOI:** 10.1038/s41523-023-00552-z

**Published:** 2023-05-31

**Authors:** Lili Chen, Fan Wu, Xiaobin Chen, Yazhen Chen, Lin Deng, Qindong Cai, Long Wu, Wenhui Guo, Minyan Chen, Yan Li, Wenzhe Zhang, Xuan Jin, Hanxi Chen, Qian Nie, Xiong Wu, Yuxiang Lin, Chuan Wang, Fangmeng Fu

**Affiliations:** 1 Department of Breast Surgery, Fujian Medical University Union Hospital, 350001 Fuzhou, Fujian Province China; 2 Department of General Surgery, Fujian Medical University Union Hospital, 350001 Fuzhou, Fujian Province China; 3 Breast Cancer Institute, Fujian Medical University, 350001 Fuzhou, Fujian Province China; 4 Department of Medical Oncology, Clinical Oncology School of Fujian Medical University, Fujian Cancer Hospital, Fuzhou, People’s Republic of China; 5 Department of Breast Surgery, Zhangzhou Affiliated Hospital of Fujian Medical University, 363000 Zhangzhou, Fujian P.R. China; 6 Department of Oncology, No. 900 Hospital of The Joint Logistic Support Force, Fuzhou, China; 7 Department of Pathology, Fujian Medical University Union Hospital, Fuzhou, China

**Keywords:** Breast cancer, Targeted therapies

## Abstract

While overweight/obesity has become a major public health issue worldwide, any association between body mass index (BMI) and therapeutic response in neoadjuvant targeted therapy treated HER2 positive breast cancer patients remain unclear. The information from a total of four-hundred and ninety-one neoadjuvant targeted therapy treated HER2 positive breast cancer patients from four institutions were retrospectively collected. Univariate and multivariate logistic analysis was developed to determine the association between BMI and therapeutic response. A meta-analysis of published literature was then conducted to confirm the effect of overweight/obesity on pCR for patients treated with neoadjuvant targeted therapy. Restricted cubic spline (RCS) adjusted for confounding factors demonstrated a decrease pCR with increasing BMI (OR = 0.937, *P* = 0.045). Patients were then categorized into under/normal weight (*n* = 299) and overweight/obesity (*n* = 192). Overweight/obese patients were independently associated with a poor therapeutic response. In the subgroup analysis, a significant negative impact of overweight/obesity on pCR can be observed both in single-targeted (OR = 0.556; *P* = 0.02) and dual-targeted (OR = 0.392; *P* = 0.021) populations. Six eligible studies involving 984 neoadjuvant targeted therapy treated HER2 positive breast cancer patients were included in the meta-analysis. The meta-analysis also demonstrated that overweight/obesity was significantly associated with a poor response to neoadjuvant anti-HER2 therapy (OR = 0.68; *P* = 0.007). Our result show that overweight and obese HER2 positive breast cancer patients are less likely to achieve pCR after neoadjuvant targeted therapy.

## Introduction

Overweight/Obesity is conventionally defined by the body mass index (BMI), characterized by abnormal or excessive accumulation of body fat, and has become a major public health issue worldwide^1^. It is widely accepted that overweight/obesity is associated with the development of breast cancer, as well as influencing its prognosis, particularly in hormone receptor (HR) positive postmenopausal breast cancer^2–4^. Although the underlying mechanism remains obscure, several studies have proposed that the chronic inflammatory state, circulating level of adipokines, insulin, insulin-like growth factor (IGF), and sex hormones might mediate the connection between overweight/obesity and breast cancer^5^.

Neoadjuvant therapy is increasingly used for treating patients with breast cancer^6^. A pathologic complete response (pCR) after neoadjuvant therapy can serve as an indicator of individual long-term survival^7^. Exploring the impact of BMI on pCR after neoadjuvant therapy provides an opportunity to assess whether BMI affects the therapeutic response in vivo, and more importantly, it might be informative for the association between BMI and long-term prognosis in breast cancer^8^. Irrespective of the potential mechanisms, there have been numerous studies committed to unraveling the relationship between BMI and the neoadjuvant therapeutic response. A recently published meta-analysis reports a negative impact of high BMI on pCR^8^. Whereas some studies suggest no association, or even demonstrate a positive effect of obesity on the pCR^9,10^. Notably, these studies included all molecular subtypes of breast cancer, which might lead to paradoxical conclusions.

Among HER2 positive patients, exploratory analysis of the NeoALTTO trial have demonstrated a borderline significant decreased pCR rate for overweight/obese patients with HR + /HER2+ breast cancer (OR = 0.55, *P* = 0.053), but not in HR-/HER2+ breast cancer (OR = 1.3, *P* = 0.331)^11^. While the subgroup analysis from a pooled study of eight prospective trials conducted by Fontanella et al. reported no association between BMI and pCR among HER2 positive breast cancer patients^12^. Currently, chemotherapy plus trastuzumab ± pertuzumab has become the standard preoperative therapy for high risk HER2 positive breast cancer. However, few studies have specifically focused on this population, rendering an unmet need to reveal the impact of BMI on pCR for HER2 positive patients treated with standard neoadjuvant therapy.

Herein, we report a retrospective multi-center analysis to determine the impact of BMI on the response to neoadjuvant targeted therapy for HER2 positive breast cancer. A meta-analysis of published studies was carried out to further validate the association between overweight/obesity and pCR for patients treated with neoadjuvant targeted therapy.

## Results

### Baseline characteristics

A total of 491 patients with a median BMI of 23.04 were included in our study. Their baseline characteristics arranged by BMI category are listed in Table 1. Among these patients, 299 (60.9%) patients were categorized into the under/normal weight group, while 192 (39.1%) cases were classified into overweight/obesity. Overweight/obesity was associated with a higher age. A significant correlation between BMI category and pCR was also observed (*P* = 0.008). No significant association was found between the BMI category and other factors.

**Table 1:** Patient characteristics by BMI categories.

Characteristics	Overall (*n* = 491)	BMI categories		*P* value^a^
		Under-/normal weight	Overweight/obesity	
		(*n* = 299)	(*n* = 192)	
Age (years)				0.002
<49	239	162	77	–
≥49	252	137	115	–
Menopausal status				0.098
Premenopausal	291	188	103	–
Postmenopausal	195	109	86	–
Unknown	5	2	3	–
Clinical T stage				0.104
T1-2	372	219	153	–
T3-4	119	80	39	–
Nodal status				0.452
Negative	111	71	40	–
Positive	380	228	152	–
Hormone receptor				0.132
Negative	240	138	102	–
Positive	251	161	90	–
HER2 staining intensity				0.425
2+	40	22	18	–
3+	451	277	174	–
Ki67 (%)				0.943
<40	203	124	79	–
≥40	288	175	113	–
Neoadjuvant targeted therapy				0.676
Trastuzumab	358	216	142	–
Trastuzumab plus Pertuzumab	133	83	50	–
Pathological response				0.008
Non-pCR	302	170	132	–
pCR	189	129	60	–

^a^*P*-values were calculated using the two-tailed *χ*^2^ test.

### BMI is associated with therapeutic response as a continuous or categorical variable

**Fig. 1:**
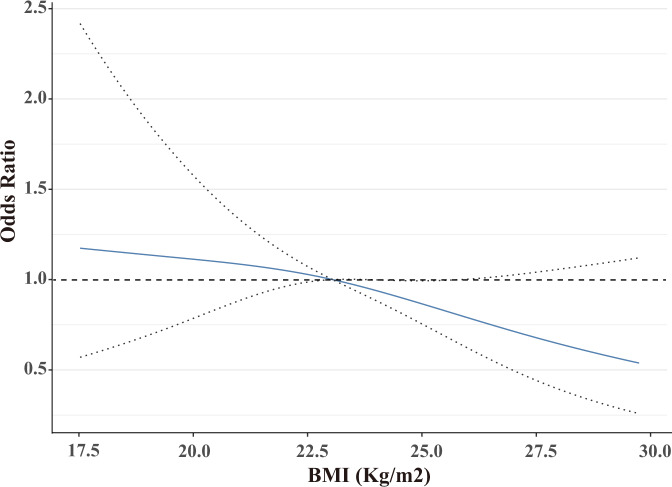
Restricted cubic splines describing the non-linear association between BMI and pCR among neoadjuvant targeted therapy treated HER2 positive breast cancer patients. Odds ratios are based on Logistic regression adjusted for age, clinical T stage, hormone receptor, HER2 staining intensity, Ki67, and neoadjuvant targeted therapy. The dotted lines represent the 95% confidence intervals for the spline model.

Overall, 189 (38.5%) patients achieved a pCR after neoadjuvant targeted therapy. A restricted cubic spline with 3-knots was developed to account for the impact of BMI on the neoadjuvant response as a continuous variable. After adjusting for potential confounders (*P* < 0.1 in univariate analysis) (Supplementary Table 1), RCS showed that a patient is less likely to achieve a pCR as the BMI increases (Fig. 1). Indeed, BMI was significantly associated with pCR as a continuous variable after adjusted by confounding factors (OR = 0.937, *P* = 0.045; data not shown).

The association between the BMI and the therapeutic response was further investigated by using BMI as a categorical variable. The pCR rates of the under/normal weight, overweight/obesity groups were 43 and 31%, respectively. Patients in the overweight/obese groups were independently associated with a poor pCR rate compared to the under-/normal weight groups in multivariate analysis (OR = 0.497, *P* = 0.001; Table 2). Collectively, our analysis suggests that BMI is correlated with therapeutic response in neoadjuvant targeted therapy treated HER2 positive breast cancer patients.

**Table 2:** Multivariate analysis of the association between BMI categories and pCR.

Characteristics	Adjusted multivariate analysis		
	OR	95%CI	*P* value^a^
Age (years)			
<49	1	–	–
≥49	1.52	1.015–2.278	0.042
BMI categories			
Under-/normal weight	1	–	–
Overweight/obesity	0.497	0.328–0.753	0.001
Clinical T stage			
T1-2	1	–	–
T3-4	0.663	0.411–1.069	0.092
Hormone receptor			
Negative	1	–	–
Positive	0.359	0.240–0.538	<0.001
HER2			
2+	1	–	–
3+	2.513	1.023–6.174	0.045
Ki67 (%)			
<40	1	–	–
≥40	1.303	0.871–1.949	0.198
Neoadjuvant targeted therapy			
Trastuzumab	1	–	–
Trastuzumab plus Pertuzumab	2.397	1.547–3.715	<0.001

^a^Two-sided *P* values were calculated using a multivariate logistic regression model.

### Association between overweight/obesity and pCR in subgroup analysis

**Fig. 2:**
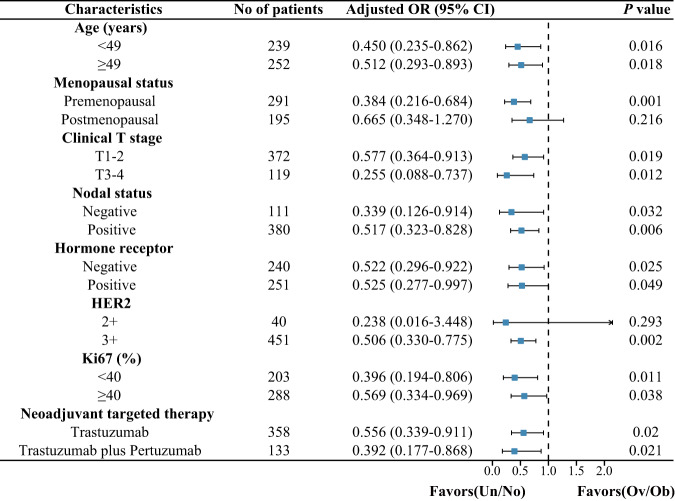
Odds ratio of pCR for all 491 patients with HER2 positive breast cancer according to BMI category in different subgroups stratified by clinical parameters (adjusted for age, clinical T stage, hormone receptor status, HER2 staining intensity, Ki67, and neoadjuvant targeted therapy, exception for stratification factor). Two-sided *P* values were calculated using a multivariate logistic regression model. Un/Nor under/normal weight, Ov/Ob overweight/obesity.

A subgroup analysis was conducted to further explored the implication of BMI in HER2 positive breast cancer patients. As shown in Fig. 2, overweight/obesity was independently associated with a poor response to neoadjuvant targeted therapy despite the patient’s age, tumor size, nodal status, hormone receptor status, Ki67 expression, or neoadjuvant targeted therapy. We also observed a statistically significant lower pCR with the overweight/obesity group for the premenopausal and HER2 3+ populations. Overweight/obese patients are less likely to achieve pCR among postmenopausal and HER2 2+ populations, although in our study this did not reach statistical significance. Overall, our subgroup analysis demonstrated a consistent trend as in the main analysis.

### Meta-analysis of the implication of overweight/obesity on pCR for HER2 positive breast cancer

**Fig. 3:**
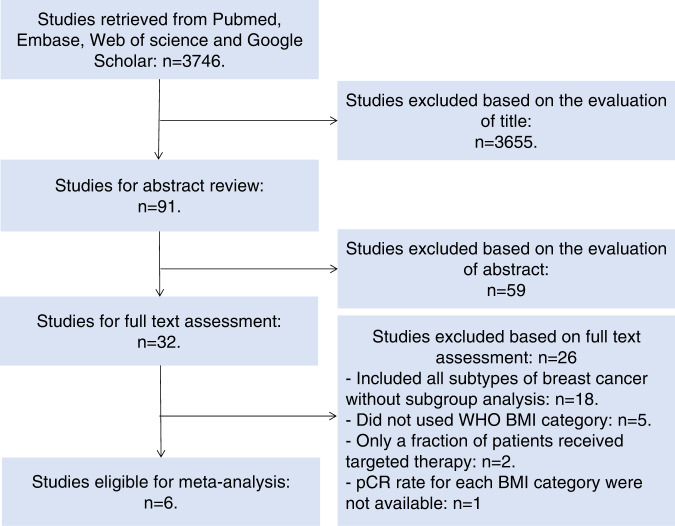
Flowchart for selection of published literature in the meta-analysis.

The literature search identified 32 studies eligible for full text review. Only six studies met the inclusion criteria and were included in our meta-analysis. Among these studies only one study was derived from a prospective randomized clinical trial^11^, the remaining five studies were retrospective^13–17^. The flowchart of our literature search and the characteristics of eligible studies are shown in Fig. 3 and Table 3.

**Table 3:** Characteristics of the eligible study.

Title	Author, year	Country	Study design	Total cases	Treatment	pCR definition	BMI category and comparison
Predictive factors of pathologic complete response in HER2- positive and axillary lymph node positive breast cancer after neoadjuvant paclitaxel, carboplatin plus with trastuzumab	Ding et al.^15^	China	Retrospective study	88	NAC+Trastuzumab	ypT0/is, ypN0	>25 vs ≤25
Stromal lymphocyte infiltration after neoadjuvant chemotherapy is associated with aggressive residual disease and lower disease-free survival in HER2-positive breast cancer	A-S. Hamy^16^	France	Retrospective study	144	NAC+Trastuzumab	ypT0/is, ypN0	<19 vs 19-25; >25 vs 19-25
Insulin-like growth factor-1, metabolic abnormalities, and pathological complete remission rate in HER2-positive breast cancer patients receiving neoadjuvant therapy	Tong et al.^14^	China	Retrospective study	81	NAC+Trastuzumab	ypT0/is, ypN0	≥ 25 vs <25
Effect of body mass index on response to neo-adjuvant therapy in HER2-positive breast cancer: an exploratory analysis of the NeoALTTO trial	Di Cosimo et al.^11^	Italy	Prospective study	454	NAC+Trastuzumab; NAC+lapatinib; NAC+Trastuzumab+lapatinib	ypT0/is	<18.5 vs 18.5 ≤ BMI < 25; ≥ 25 vs 18.5 ≤ BMI < 25; ≥ 30 vs 18.5 ≤ BMI < 25
Carboplatin dose capping affects pCR rate in HER2‐positive breast cancer patients treated with neoadjuvant Docetaxel, Carboplatin, Trastuzumab, Pertuzumab (TCHP)	Howell et al.^13^	UK	Retrospective study	124	NAC+Trastuzumab+Pertuzumab	ypT0/is, ypN0	≥ 25 vs <25
Clinical verification of body mass index and tumor immune response in patients with breast cancer receiving preoperative chemotherapy	Takada et al.^17^	Japan	Retrospective study	93	NAC+Trastuzumab	ypT0/is, ypN0	>18.5 vs ≤18.5; >25 vs ≤25; >30 vs ≤30

*NAC* neoadjuvant chemotherapy.

**Fig. 4:**
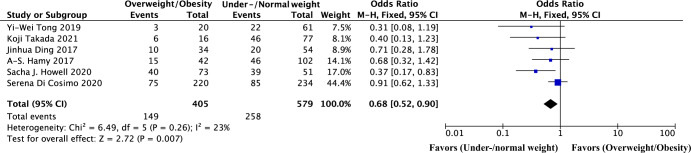
Forest plot of odds ratio for pCR in overweight/obesity vs under/normal weight among patients with HER2 positive breast cancer treated with neoadjuvant targeted therapy.

All six studies provided data that divided patients into two groups (BMI < 25 kg/m^2^ and ≥25 kg/m^2^), hence, our pooled analysis investigated the impact of overweight/obesity (BMI ≥ 25 kg/m^2^) on the neoadjuvant therapy response versus under/normal weight patients (BMI < 25 kg/m^2^). A total of 984 HER2 positive breast cancer patients treated with anti-HER2 based neoadjuvant therapy from six studies were included in our meta-analysis. We found a significant decrease in the pCR in overweight/obese patients compared to the under/normal weight patients (OR = 0.68, *P* = 0.007; Fig. 4), consistent with our results.

## Discussion

In the present study, we surveyed data from 491 patients and found that overweight/obesity was associated with a lower pCR rate in HER2 positive breast cancer patients after neoadjuvant targeted therapy. In addition, a meta-analysis of published literature was conducted, and it further confirmed the negative influence of BMI on the pCR in a targeted therapy treated HER2+ breast cancer population.

Substantial efforts have been made to investigate the influence of BMI on the therapeutic response of breast cancer patients, but there remains controversy. A recently published meta-analysis revealed that overweight/obese patients had a lower pCR rate compared to under/normal weight patients^8^. This result was consistent with several studies^18,19^. However, some studies have offered conflicting perspectives^9,20^, but these studies did not focus on a specific subtype of breast cancer. Breast cancer is a heterogeneous disease with different subtypes and the mechanism of carcinogenesis, and the treatment strategies vary among these subtypes^21^. The BMI may play a diverse role in the neoadjuvant therapeutic response depending on the subtype of breast cancer, and this might explain the conflicting results from previous studies.

In our study, we found an independent negative impact of BMI on pCR in HER2 positive breast cancer patients treated with neoadjuvant targeted therapy. The underlying molecular mechanisms by which overweight/obesity could influence the effect of targeted therapy are currently unclear. Sex hormones have been reported to mediate cross talk between overweight/obesity and breast cancer due to the endocrine role of adipose tissue, especially in postmenopausal women, even though BMI does not measure adiposity directly^4,5,22,23^. Indeed, analysis of the NeoALTTO trial shows a tendency towards a lower pCR rate for the overweight/obesity patients compared to the under/normal weight patients in HR + /HER2+ (OR = 0.56, *P* = 0.054), but not in HR-/HER2+ (OR = 1.31, *P* = 0.324) breast cancer patients^11^. Interestingly, a similar effect of overweight/obesity on pCR was demonstrated in HR-/HER2+ (OR = 0.522, *P* = 0.025) and HR + /HER2+ (OR = 0.525, *P* = 0.049) breast cancer patients in our analysis. We also observed a significant negative impact of BMI on pCR in premenopausal patients. This suggests that other mechanisms rather than sex hormones are involved.

The tumor immune microenvironment (TIME) plays an important role in the therapeutic response in HER2 positive breast cancer. The impact of tumor immunity is important for patients treated with targeted therapy since the effect of trastuzumab and pertuzumab is partially reliant on antibody-dependent cellular cytotoxicity (ADCC)^24^. It has been reported that adiposity accumulation can result in an immunosuppressive TIME^25^. For instance, overweight/obesity-related increases in the chemokine CCL2, estrogen, and pro-inflammatory mediators could induce the accumulation of myeloid-derived suppressor cells (MDSCs) in breast cancer tumors^11,26^. In addition, a lower density of tumor-infiltrating lymphocytes (TILs) has been reported in overweight/obese HER2+ breast cancer patient tumors compared to tumors from normal weight counterparts^17^. The immunosuppressive status of the TIME might contribute to the poor targeted therapy response in overweight/obese HER2 positive breast cancer patients.

In vitro and in vivo studies have suggested that adipose cells promote the resistance to trastuzumab-mediated ADCC via the secretion of soluble factors, despite the mechanism remaining unclear^27^. The immunological function of leptin has recently attracted much interest, especially for the impact of leptin on natural killer (NK) cells – one of the most crucial mediators of ADCC^28,29^. A short-term stimulation of leptin could result in a significant NK cell functional activation. However, chronic exposure to an elevated concentration of leptin in overweight/obesity might decrease the anti-tumor immune response of NK cell by inhibiting NK cell functions^30^. In addition to the immunological role of leptin, the crosstalk between leptin signaling and HER2 signaling might also reduce sensitivity to targeted therapy, thus lead to a low pCR rate^31^.

Overweight/obese patients tend to have an increased concentration of circulating insulin-like growth factors-1 (IGF-1) that can result in an overactivation of insulin-like growth factors-1 receptor (IGF-1R) signaling^32,33^. Preclinical studies have revealed that the aberrant activation of IGF-1R signaling promotes cell survival via the PI3K-AKT-mTOR and RAS-RAF-MAPK pathways and contribute to the poor response to anti-HER2 therapy^34^. The association between circulating IGF-1 and pCR has been investigated by Tong et al. in a retrospective clinical study, where they found that a low IGF-1 level was related with a higher pCR rate (OR = 3.93, *P* = 0.031) in trastuzumab treated HER2 positive breast cancer patients^14^. Therefore, elevated circulating IGF-1 levels in overweight/obese patients might contribute to the inferior targeted therapy response in this population.

The pharmacokinetics of monoclonal antibodies associated with obesity might also be involved in the poor response to targeted therapy. It has been demonstrated that higher body weight of patients is associated with increased trastuzumab or pertuzumab clearance, which might lead to a lower plasma concentration in overweight/obese patients^35,36^. It is hard to determine the relationship between the trastuzumab plasma concentration and neoadjuvant therapeutic response due to the lack of relevant data. However, a lower trastuzumab concentration has been observed in metastatic gastric cancer patients with progressive disease than in those with partial or complete response or stable disease^37^. These findings might bring new insights, although it might not be appropriate to extrapolate the conclusion from gastric cancer to breast cancer^38^.

Our study has several limitations. The first limitation is the retrospective nature of the study. Secondly, Asians at a given BMI have a higher percentage of body fat than White or European populations^39^. Due to the variation in the body composition across regions and ethnicities, we defined overweight/obesity according to the Chinese WGOC definition in our multi-center study, since it was derived from a large Chinese population^40^. However, WHO criteria were employed in the subsequent meta-analysis. The differences of definition in overweight/obesity between multi-center study and meta-analysis might lead to a difficulty in the interpretation of our research. Finally, we could not explore the association between BMI and the prognosis of HER2+ patients due to the lack of prognostic information.

In conclusion, based on a multi-center retrospective study and meta-analysis, we found a negative impact of overweight/obesity on the therapeutic response for HER2 positive breast cancer patients treated with neoadjuvant targeted therapy. Further studies are needed to shed light on the complex mechanism behind this phenomenon.

## Methods

### Patient selection and data acquisition

Information from a total of 491 patients diagnosed with HER2 positive breast cancer, all of whom had undergone neoadjuvant targeted therapy followed by surgery between June 2012 to December 2021, was consecutively collected from four institutions (Fujian Medical University Union Hospital, Fujian Cancer Hospital, Zhangzhou Affiliated Hospital of Fujian Medical University, and No. 900 Hospital of The Joint Logistic Support Force). Breast cancer diagnosis was confirmed by core needle biopsy prior to neoadjuvant therapy. Neoadjuvant regimens consisted of: 1) EC-T plus targeted therapy (epirubicin 100 mg/m^2^ and cyclophosphamide 600 mg/m^2^ every three weeks for four cycles followed by docetaxel 80 mg/m^2^ and targeted therapy every three weeks for four cycles), 2) TCb plus targeted therapy (docetaxel 75 mg/m^2^, carboplatin [area under curve = 6] and targeted therapy every three weeks for six cycles), 3) THP (docetaxel 80 mg/m^2^, trastuzumab initiated with a loading dose of 8 mg/kg followed by a maintenance dose of 6 mg/kg, and pertuzumab 840 mg as a loading dose at cycle 1 and 420 mg thereafter every three weeks for four cycles). Trastuzumab or trastuzumab plus pertuzumab was given for patients who received targeted therapy. Patients with dose reduction were excluded from the present study.

Clinicopathological characteristics of participants were collected, including demographic information, imaging examination, pathological evaluation of biopsy and surgical specimens, and treatment records. A median age of 49 was employed to classify patients into two groups. Women were considered postmenopausal if they had no menstruation in the past 12 months. Clinical tumor size and axillary lymph node status before neoadjuvant therapy was evaluated by color Doppler ultrasound and was defined in accordance with American Joint Committee on Cancer (AJCC) breast cancer staging manual 8th. Patients with metastatic disease, and those with unavailable baseline BMI were excluded from the study. The protocol of this study was approved by the ethics committee of Fujian Medical University Union Hospital, ethics committee of Fujian Cancer Hospital, ethics committee of Zhangzhou Affiliated Hospital of Fujian Medical University, and ethics committee of No. 900 Hospital of The Joint Logistic Support Force. All participants gave their written informed consent before their inclusion.

### BMI calculation and categorization

The height and weight of patients was recorded within one week prior to receiving neoadjuvant therapy in their first hospitalization. BMI was calculated as the weight (Kg) divided by the square of the height (m^2^) and was used to defined obesity in our study. Due to the variation in the body composition across regions and ethnicities, the WGOC definition derived from a large Chinese population was employed to categorize patients into underweight (BMI < 18.5 kg/m^2^), normal weight (18.5 to <24 kg/m^2^), overweight (24 to <28 kg/m^2^), and obese (≥28 kg/mg^2^)^39,40^. The impact of BMI on the neoadjuvant therapeutic response was investigated as a continuous or categorical variable.

### Pathological evaluation

Estrogen receptor (ER), progesterone receptor (PR), and HER2 were evaluated by immunohistochemistry (IHC). Patients were defined as positive for ER or PR if at least 1% of the tumor nuclei were stained, and they were hormone receptor (HR) negative when both ER and PR were found to be negative. HER2 was defined as positive when IHC results were 3+ or 2+ with HER2 amplification evaluated by fluorescence in situ hybridization (FISH)^41^. A median Ki67 index (by IHC) of 40% was used to divide patients into a low Ki67 index group (<40%) and a high Ki67 index group (≥40%). A pCR was defined as the absence of residual invasive disease in the breast and axillary lymph nodes (ypT0/is, ypN0).

### Literature review and meta-analysis

A systematic literature search and meta-analysis was performed to further investigate the implications of BMI on neoadjuvant targeted therapy of HER2 positive breast cancer patients. Relevant research published prior to December 1, 2021, was retrieved from Pubmed, Embase, Web of Science, and Google Scholar. The key search terms were “breast neoplasms” or “breast cancer”, “neoadjuvant therapy” or “neoadjuvant” or “pathologic complete response”, “trastuzumab” or “molecular targeted therapy”, “body mass index” or “obesity” or “overweight”. Literature retrieval was also conducted by reviewing the references of reviewed studies. Studies were eligible for inclusion if they; (1) had available data of the distribution of the BMI category and corresponding pCR rate in HER2 positive breast cancer, (2) neoadjuvant targeted therapy was given for HER2 positive breast cancer, and (3) pCR was defined as no residual invasive cancer in breast or nodes with residual noninvasive breast cancer allowed (ypT0/is ypN0), or the absence of invasive cancer cells in the breast irrespective of the presence of lymph node infiltration by malignant cells (ypT0/is). Two authors independently performed the literature search, study screening, and data extraction.

### Statistical analysis

A chi-square test was employed to explore the association between the BMI categories and the following clinicopathological characteristics: age, menopausal status, clinical T stage, Nodal status, hormone receptor status, HER2 staining intensity, Ki67, neoadjuvant targeted therapy, and pathological response. An RCS with 3-knots was used to allow the investigation of a non-linear association between BMI and pCR. Univariate logistic analysis was used to assess factors correlated with the pCR, factors with a *P* < 0.1 in univariate analysis were included in multivariate analysis (menopausal status was excluded from multivariate analysis due to the high consistency between menopausal status and age). All statistical analyses were performed by R (version 4.0.0) and SPSS (Version 26.0).

The meta-analysis was performed by Review Manager 5.4. Heterogeneity was assessed by *I*^2^ statistics, and an *I*^2^ < 50% indicated there was no heterogeneity among these studies, and a fixed effect model was used in meta-analysis. Otherwise, a random effect model was applied. We conducted a funnel plot to assess publication bias. If the funnel plot was symmetric, then there was no publication bias. *P* values were significant at *P* < 0.05.

### Reporting summary

Further information on research design is available in the Nature Research Reporting Summary linked to this article.

## Supplementary information

Supplementary Table 1

Reporting Summary

## Data Availability

Data were available from corresponding authors upon reasonable request.
