# Supplementary material for: Impact of body mass index in therapeutic response for HER2 positive breast cancer treated with neoadjuvant targeted therapy: a multi-center study and meta-analysis
Source: NPJ Breast Cancer. 2023 May 31;9:46. doi: 10.1038/s41523-023-00552-z (PMC10232579; doi:10.1038/s41523-023-00552-z)
Supplement: Supplementary file 1 — Supplementary Table 1 [file 41523_2023_552_MOESM1_ESM.pdf]

Supplementary Table 1. Univariate analysis of the relationship between clinicopathological characteristics and pCR.

	Unadjusted univariate analysis		
**Characteristics**	**OR**	**95%CI**	***P* value***
**Age (years)**			
<49	1		
≥49	1.625	1.125-2.346	0.01
**Menopausal status**			
Premenopausal	1		
Postmenopausal	1.389	0.959-2.013	0.082
**BMI categories**			
Under-/Normal weight	1		
Overweight/Obesity	0.599	0.409-0.877	0.008
**Clinical T stage**			
T1-2	1		
T3-4	0.653	0.421-1.014	0.058
**Nodal status**			
Negative	1		
Positive	1.333	0.855-2.080	0.205
**Hormone receptor**			
Negative	1		
Positive	0.365	0.251-0.532	<0.001
**HER2**			
2+	1		
3+	3.19	1.381-7.366	0.007
**Ki67 (%)**			
<40	1		
≥40	1.438	0.990-2.090	0.057
**Neoadjuvant targeted therapy**			
Trastuzumab	1		
Trastuzumab plus Pertuzumab	2.431	1.619-3.650	<0.001

* Two-sided *P* values were calculated using a univariate logistic regression model.
